# Quantifying uncertainties of sandy shoreline change projections as sea level rises

**DOI:** 10.1038/s41598-018-37017-4

**Published:** 2019-01-10

**Authors:** Gonéri Le Cozannet, Thomas Bulteau, Bruno Castelle, Roshanka Ranasinghe, Guy Wöppelmann, Jeremy Rohmer, Nicolas Bernon, Déborah Idier, Jessie Louisor, David Salas-y-Mélia

**Affiliations:** 1 BRGM, 3, av. Claude Guillemin, BP 36009, 45060 Orleans Cedex 2, France; 2 BRGM, French Geological Survey, Pessac, France; 3 CNRS/Univ. Bordeaux, Pessac, France; 4 IHE Delft/University of Twente/Deltares, Delft, The Netherlands; 5 LIENSs, CNRS - Université de La Rochelle, La Rochelle, France; 6 CNRM, Université de Toulouse, Météo-France, CNRS, Toulouse, France

## Abstract

Sandy shorelines are constantly evolving, threatening frequently human assets such as buildings or transport infrastructure. In these environments, sea-level rise will exacerbate coastal erosion to an amount which remains uncertain. Sandy shoreline change projections inherit the uncertainties of future mean sea-level changes, of vertical ground motions, and of other natural and anthropogenic processes affecting shoreline change variability and trends. Furthermore, the erosive impact of sea-level rise itself can be quantified using two fundamentally different models. Here, we show that this latter source of uncertainty, which has been little quantified so far, can account for 20 to 40% of the variance of shoreline projections by 2100 and beyond. This is demonstrated for four contrasting sandy beaches that are relatively unaffected by human interventions in southwestern France, where a variance-based global sensitivity analysis of shoreline projection uncertainties can be performed owing to previous observations of beach profile and shoreline changes. This means that sustained coastal observations and efforts to develop sea-level rise impact models are needed to understand and eventually reduce uncertainties of shoreline change projections, in order to ultimately support coastal land-use planning and adaptation.

## Introduction

Since 1870, sea level has been rising, mainly due to the melting of land-ice and ocean expansion caused by anthropogenic climate warming^1–5^. While the most immediate impact of sea-level rise is increased coastal flooding hazards^6–13^, there are significant concerns regarding shoreline retreat as well^6,14–26^. In particular, beaches backed by sandy deposits are receiving particular attention for several reasons: first, they represent 31% of the world’s ice-free coasts^25^; second, they are potentially highly sensitive to sea-level changes^14–16,24^; third, it has been estimated that at least 24% and up to 70% of the world beaches are already under chronic erosion, although with large regional and local differences^25,27^; finally, beaches are both valuable for tourism and as buffer zones during extreme events such as storms.

Sandy shoreline change projections along a given coast need to consider sediment losses and gains caused by a number of hydro-sedimentary processes acting at different timescales^28^. Coastal change is driven by a myriad of processes interacting with one another at a wide range of spatial and temporal scales, making the use of process-based coastal evolution modelling difficult at operational levels. Hence, instead of attempting to quantify the impact of each process causing sediment transport, coastal adaptation practitioners have pragmatically relied on extrapolations of past observations in order to anticipate future shoreline changes. In the absence of human interventions, estuaries or other major sediment sources or sinks, this results in the following equation:

1 $${L}_{r}={L}_{r0}+n.Tx+Lvar+\Delta {S}_{slr}$$

where ${L}_{r0}$ and ${L}_{r}$ are the shoreline positions in a cross-shore direction at the initialization and after $n$ years, respectively, $Tx$ is the linear trend over multi-decadal to centennial timescales, and $Lvar$ represents the seasonal, inter-annual and decadal modes of variability of shoreline changes. Neither $Tx$ nor $Lvar$ is related to a single physical process: for example, the effects of longshore gradients in sediment transport are included in $Lvar$ when they result in evolution and cycles occurring at interannual to decadal timescales, while there are included in $Tx$ if their manifestation involves longer timescales^29^. Finally, the term $\Delta {S}_{slr}$ quantifies the impacts of relative sea-level changes, that is, those due to ocean thermal expansion, land water and ice contributions, and vertical ground motions (uplift or subsidence)^30^. This is denoted “coastal impact model” hereafter.

In the area of coastal prospective planning, a comprehensive description of the uncertainties of future shoreline positions is required in order to avoid maladaptation^31^. Shoreline change projections based on equation (1) need to consider the uncertainties of future sea-level rise under different climate forcings^2,32^, of local vertical ground motions^33^, of other modes of variability of shoreline positions ($Tx$ and $Lvar$), as well as uncertainties of coastal impact models ($\Delta {S}_{slr}$). The latter source of uncertainty has not been quantified so far.

We estimate the uncertainties of coastal impact models ($\Delta {S}_{slr}$) by considering the difference between two existing approaches: the Bruun rule^34^ and the Probabilistic Coastline Recession model^35–39^. The Bruun rule is the most commonly used and historical approach to assess sea-level rise impacts on shorelines^34,40,41^. It assumes a landward translation of the beach profile as sea level rises. The Probabilistic Coastline Recession model (PCR) is a recently introduced approach that quantifies sediment losses at the dune toe during storms, as well as the nourishment of the dune by aeolian sediment transport processes between storms^35^. Over multi-decadal timescales, the superimposition of unchanged storms with rising mean sea levels results in more frequent and larger sediment losses in the PCR model. The Brunn rule and the PCR models are not only based on different assumptions regarding the physical processes guiding the response of sandy shorelines to sea-level rise, but they also provide different results^35,42^. Today, both coastal impact models are equally difficult to validate due to the scarcity of coastal data and the complexity of the hydrosedimentary processes involved^43,44^. Faced with this structural uncertainty^45^, stakeholders concerned with coastal adaptation generally lack the relevant observations to validate one particular model, and may therefore assign an equal confidence to both, following the maximum entropy principle^46^. Hence, we consider the difference between the Bruun and PCR models as a first order measure of the coastal impact model error.

Equation (1) requires coastal data, which are not available at the global scale. Hence, we implement the approach in the sandy coast of Aquitaine in southwestern France (Supplementary Materials 1, 2 and 3), where observations of shoreline positions^47^ and of other metrics allow to minimize uncertainties caused by the lack of data, thus avoiding mixing them with uncertainties due to the variability of the different processes (Supplementary Material 1, section 1). We select four coastal sites because they are representative of the broad range of variability and trends in shoreline positions in the sandy coast of Aquitaine (Supplementary Materials 1, 3 and 4): sites #1 and #2 are stable today, whereas sites #3 and #4 are eroding. The variability around this trend reaches +/−21 m at site #3, approximately +/−9 m at site #2 and #4 and only +/−3.2 m at site #1. Furthermore, site #2 is located close to a permanent Global Navigation Satellite System (GNSS) station, providing a precise evaluation of vertical ground motions (subsidence or uplift) and of its contribution to relative sea level changes. The uncertainties of vertical ground motions at site #2 are estimated from the SONEL database^48^, while those at sites #1, #3 and #4 are based on a global analysis of coastal vertical ground motions based on GNSS measurements and Global Isostatic Adjustment (GIA) modeling. Hence, site #2 provides a testbed to appraise the usefulness of GNSS instrumentation in reducing uncertainties of shoreline change reconstructions and projections.

**Figure 1:**
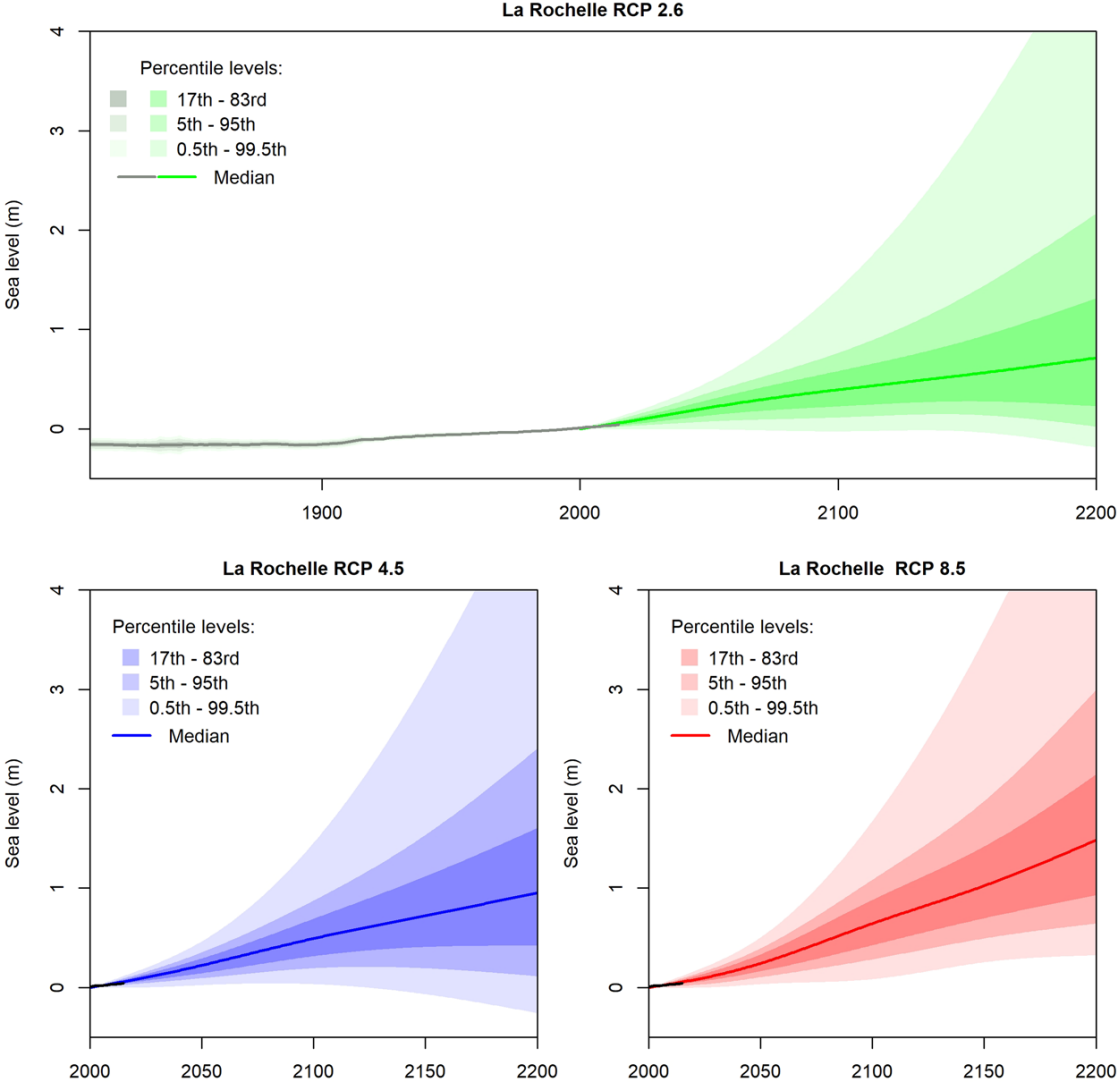
Sea level reconstructions and projections used in this study (source of projections: Kopp *et al*.^32^; Reconstruction: see Methods, subsection 1).

We compute shoreline change projections for the four coastal sites in Aquitaine by means of a Monte-Carlo procedure, within which probabilistic input parameters (Supplementary Material 2) are propagated through equation (1)^20,49^. We estimate past regional sea-level changes and their uncertainties using tide-gauge records and permanent GNSS stations providing estimates of coastal vertical ground motions^48^ (Fig. 1). We use the future sea-level rise projections provided by Kopp *et al*.^32^ that we correct from local vertical ground motions (Fig. 1; see Methods, subsection 1). These probabilistic sea-level projections^32^ are essentially consistent with the 5^th^ Assessment Report of the International Panel on Climate Change (IPCC)^2^, which remains today the reference source of information from the perspective of coastal adaptation practitioners (see the discussion section). For example, for RCP 8.5 by 2100, the 17^th^ and 83^rd^ quantiles of the probabilistic projections of Kopp *et al*.^32^ indicate a global mean sea level rise of 0.62 to 1 m. This compares well with the likely range of the IPCC projections for the same time horizon (0.52 to 0.98 m), corresponding to a 66–100% probability according to the IPCC uncertainty language, or roughly 66% probability according to a later communication of the IPCC authors^50^. The other terms in equation (1) ($Tx$ and $Lvar$) are evaluated empirically, based on past observations of ${L}_{r}$ obtained through various data (e.g. historical charts, satellite and aerial photographs, shoreline surveys) available from the Aquitaine Coastal Observatory (Supplementary Materials 1 and 2) for each coastal impact model.

We evaluate the uncertainties of coastal impact models against all other sources of uncertainties using a global sensitivity analysis^51^ (see Methods, subsection 3). This latter procedure provides quantitative estimates of the contribution of each uncertain parameter to the variability of shoreline change projections, while addressing explicitly interactions between uncertain parameters involved in equation (1). The global sensitivity analysis involves a large number of simulations: the PCR model would be run approximately 100,000 times each year to reduce errors of sensitivity indices to less than 1%. As the computation time of the PCR model is prohibitive for such a number of simulations, we use a surrogate model of the PCR model to reduce the computation time (see Methods, subsection 4 and Supplementary Material 5).

Our probabilistic approach delivers a range of contrasting shoreline change projections. We show that by 2100, depending on the location of interest, the statistical uncertainties of sea-level projections account for up to approximately half of that due to the choice of a particular coastal impact model (section 2). However, there remain residual uncertainties, the probability of which is difficult to quantify, and which could bring future shoreline positions outside the range of probabilistic projections shown in section 2 (see section 3). Despite these residual uncertainties, this study shows that the uncertainties of coastal impact models should be considered in future global studies aiming at quantifying sea-level rise impacts and their uncertainties.

## Results

### Shoreline change projections

**Figure 2:**
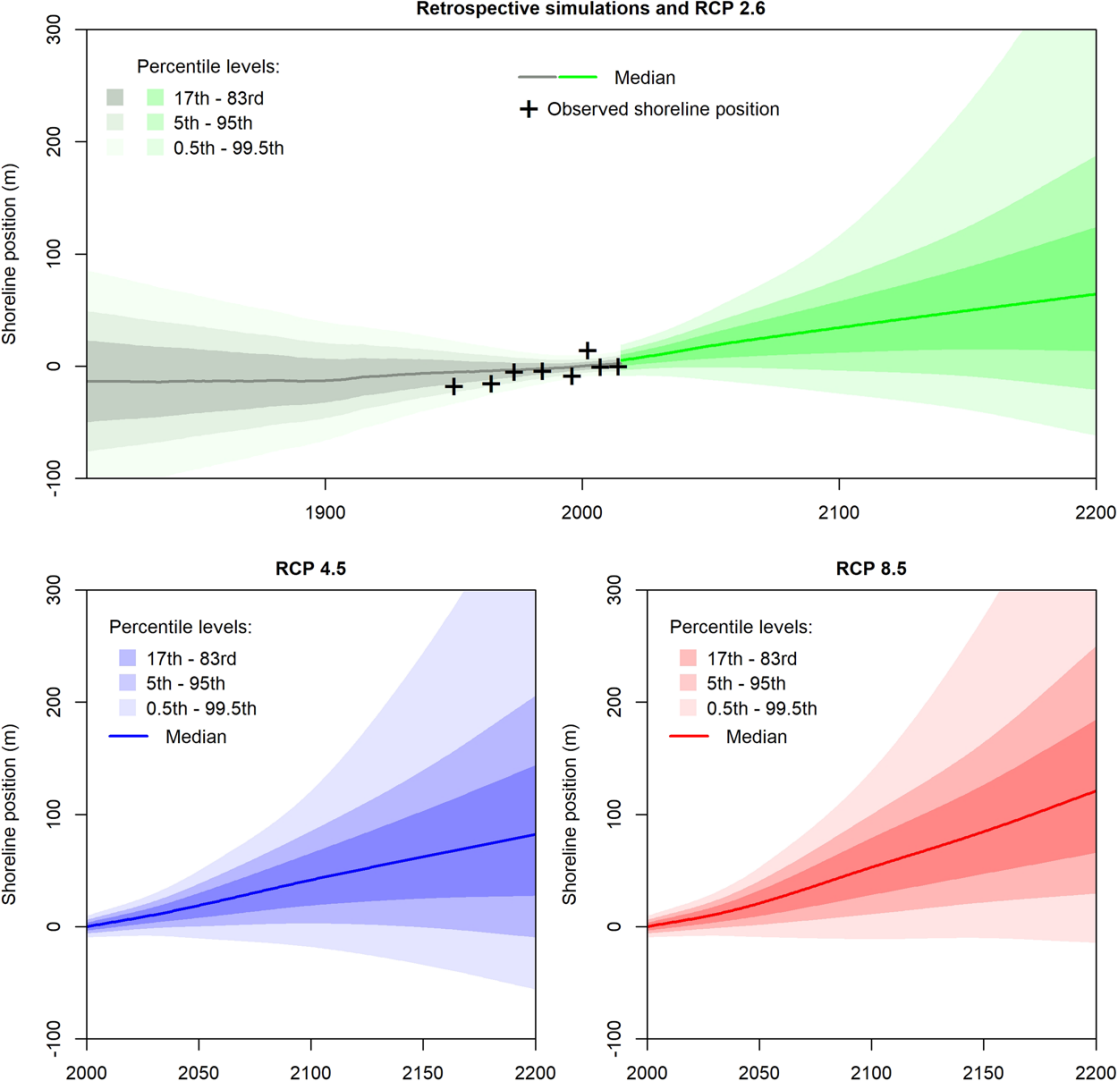
Reconstructions and projections of shoreline positions using the Bruun rule exemplified at site #1 (See Supplementary materials 1, 3 and 4), provided in the form of median and percentile levels at different timeframes from 1807 to 2200. The reference median shoreline position is arbitrarily set to 0 by 2000, with negative values corresponding to shoreline accretion (seaward) and positive to shoreline retreat (landward). These projections include uncertain shoreface slopes, vertical ground motions, sea-level changes, shoreline and change variability from event-scale to inter-decadal timescales as well as an uncertain multi-decadal trend (see Methods). Note that sea-level projections used here^32^ consider that a sea-level drop is possible (although very unlikely) beyond 2100 (Fig. 1). Hence, shoreline changes below the median can bend downwards beyond 2100.

**Figure 3:**
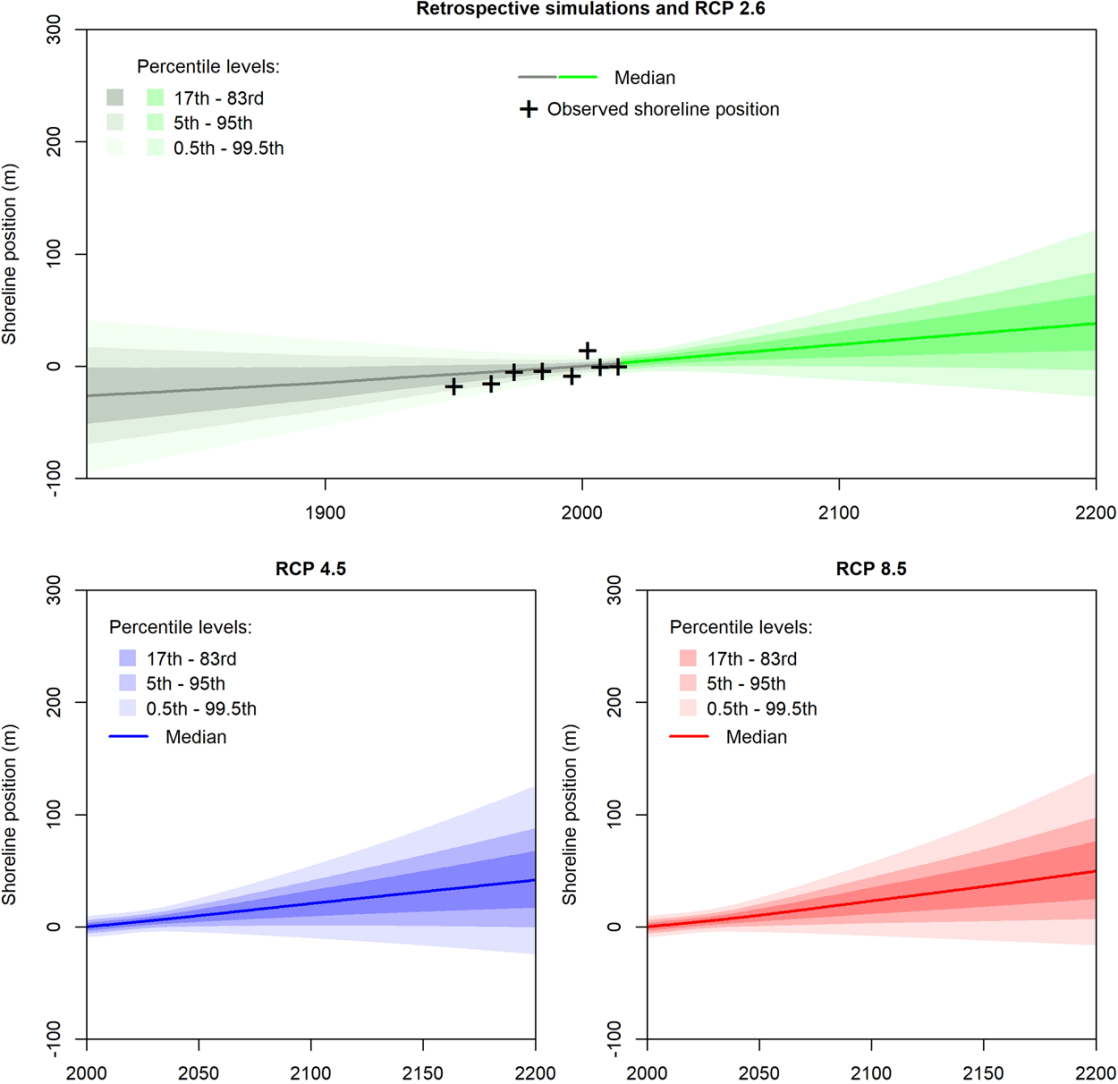
Same as Fig. 2, with shoreline change projections and reconstructions using the PCR coastal impact model.

This subsection presents the shoreline change reconstructions (1807 to 2014) and projections (2000 to 2200) obtained from the propagation of probabilistic uncertainties through equation (1) at the coastal site #1 (for other sites, see Supplementary Materials 6 to 11). We provide shoreline change projections and reconstructions based on equation (1) implementing the Bruun rule (Fig. 2) and the surrogate PCR model (Fig. 3). For consistency, the effects and uncertainties of past sea-level changes have been included in past shoreline change positions as assumed in equation 1, although they are expected to remain small^47,52^.

At each time-step from 1807 to 2200, Figs 2 and 3 provide the median shoreline positions along a cross-shore transect. In addition, the uncertainties of shoreline change projections are conveyed through percentile levels. Thus, the probability for a shoreline position to remain between the 17^th^ – 83^rd^ percentile levels (most intense gray, green, blue or red colors) is 66%. Note that according to these projections, there is a small probability that shorelines advance seaward in the future. This is because sea-level rise projections used here^32^ neither exclude a decrease in the rate of sea-level rise after some decades, nor a drop in sea-level rise itself in the case of RCP 4.5 and RCP 2.6 (Fig. 1). The uncertainties of the shoreline change projections are very large (50 to 100 m by 2200), especially for the simulations based on the Bruun rule (Fig. 2). These uncertainties are analyzed in the following section by means of the global sensitivity analysis.

All median projections shown in Figs 2 and 3 involve shoreline erosion, which corresponds to the superimposition of a multi-decadal eroding trend at site #1 with the effects of sea-level rise. Retreat values are larger for scenarios with highest radiative forcing: median values reach 40 m and 100 m for RCP 4.5 and RCP 8.5 by 2100 with the projections based on the Bruun rule. Projections based on PCR are less sensitive to sea-level rise, so that shoreline retreat values and the related statistical uncertainties are approximately five times smaller in Fig. 3 compared to Fig. 2 (note similar order of magnitudes in Supplementary Materials 6 to 11 for sites #2, #3 and #4). This agrees well with previous studies showing that by 2100, the Bruun estimate lies in the range of 4–40% exceedance probability with respect to the corresponding approach based PCR estimates^35,42^.

For coastal adaptation practitioners, such results mean that at similar coastal sites where buildings and infrastructures are located close to the coast, the modeling approach based on the Bruun rule implies large shoreline retreats for high emission scenarios in a few decades from now, superimposing a trend of 0.5 m/yr or more to current shoreline retreat rates. Such a change would be associated with expensive coastal protection measures or the relocation of numerous assets at risk. Conversely, RCP 2.6 and the PCR model generate less erosive trends, so that no specific adaptation to sea-level rise-induced shoreline erosion would be required. In all cases, a strong increase in shoreline erosion is not expected before 2050, which gives time to plan adaptation.

### Variance-based global sensitivity analysis

**Figure 4:**
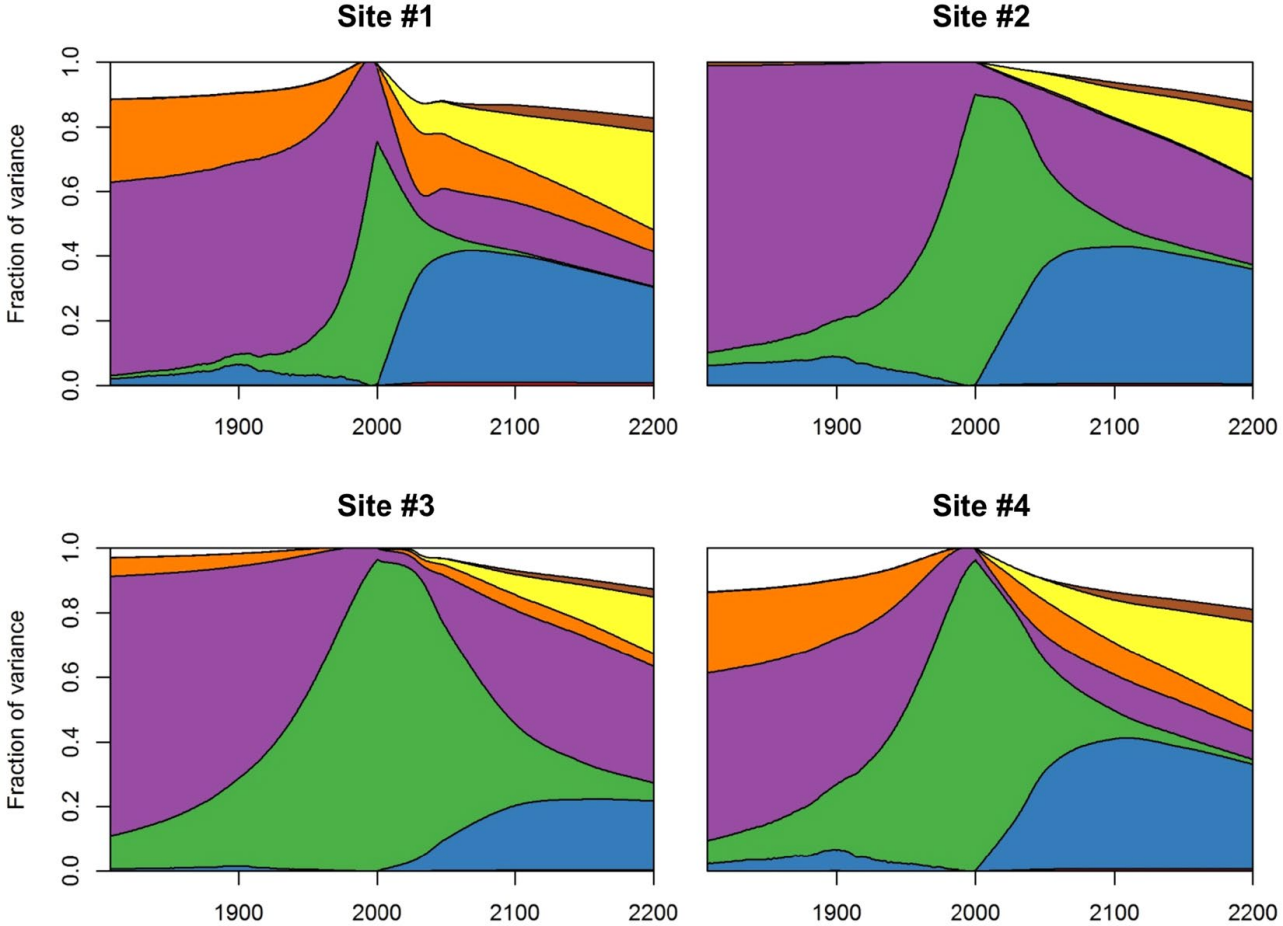
Variance-based global sensitivity analysis of the shoreline change model response as a function of time, for the four selected sites in Aquitaine (see Supplementary Material 1). For each date considered, the curves indicate the fraction of the variance of shoreline change projections that could be removed if input parameters were known (see main text). The effect of interactions between parameters is indicated as well. White areas indicate interactions between parameters, corresponding to shoreline positions, which can be only reached if at least two uncertain parameters deviate from their mean (see Methods). The graph reads as follows: for site #1, by 2200, uncertainties in regional sea-level rise projections (yellow) account for approximately 30% of the variance of shoreline change projections.

Figure 4 partitions the variance of shoreline change projections by displaying the main effects (i.e. 1^st^ order Sobol’ indices) of the uncertain parameters in equation (1). This index is commonly interpreted as the expected proportion of the total variance of the shoreline change that would be removed if we were able to learn the true value of the uncertain parameter. It is commonly used to rank the importance of model parameters according to their impact for the variability of the model outcome^51^.

The variance of shoreline change projections is partitioned differently depending on the period of time considered (Fig. 4): uncertainties in the long-term trends (not including sea-level rise, that is, $Tx$ in equation (1)) have a larger impact in the past (before 1950), whereas for present days, uncertainties are overwhelmingly due to the current modes of shoreline change variability, associated with the random nature of waves and currents at timescales ranging from days to decades through seasons ($Lvar$ in equation (1)). Whatever the coastal site and the period of time considered, the uncertainties of the regional sea-level reconstructions can be neglected with little impact to shoreline change projections, and so can the uncertainties of shoreface slopes estimations. Where vertical ground motions are known precisely from a permanent GNSS station, their impact on shoreline change projections is negligible (site #2), whereas they can account for up to almost 20% of the variance by 2050 at site #1. Hence, while all sites considered are exposed to quite similar waves and tide conditions, the relative importance of each source of uncertainty varies along the coast depending on the local processes and the knowledge available.

If we knew which coastal impact model was correct, the variance of shoreline change projections could be reduced by 20 to 40% by 2100 depending on the coastal site of interest. Indeed, Fig. 4 shows that for sites #1, #2 and #4, the impact of this source of uncertainty grows rapidly over the coming decades, then peaks at 40% during the second half of the 21^st^ century, and finally decreases after 2100, as sea-level projections are becoming more uncertain and account for a larger fraction of the variance of shoreline change projections. For site #3, the uncertainties of shoreline change trends and variability are much larger, thus reducing the fraction of variance that can be attributed to the choice of a coastal impact model. However, whatever the coastal site considered, the uncertainty due to coastal impact models accounts for twice the impact of the uncertainties of probabilistic sea-level projections (yellow area in Fig. 4).

It could be argued that although the Bruun rule and the PCR models are two common alternatives, they could both be wrong. However, if the true response of shorelines to future sea level rise is outside the range predicted by the two coastal impact models, the relative importance of this structural uncertainty will even be greater than estimated in Fig. 4.

To summarize, the main sources of uncertainties of shoreline reconstructions and projections depend on the local coastal settings and vary with time, with future sea-level rise and the choice of a particular coastal impact model becoming prominent beyond 2050. This statement relies on state-of-the-art probabilistic uncertainty quantification exercises, which, nevertheless, still contains some inherent limitations (see section 3). It is more than likely that our understanding of uncertainties will evolve as new knowledge becomes available, leading to new, better assessments and rankings of uncertainty sources. Meanwhile, the different sources of uncertainties shown in Fig. 4 can be addressed differently, either by trying to reduce them using new observations wherever possible (e.g., of shoreline changes and vertical ground motions)^53^, or by selecting appropriate decision-making frameworks to minimize the risks of maladaptation despite uncertainties^31,54^.

## Discussion: Residual Uncertainties

Shoreline change projections presented here assume that the uncertainties of each input parameter can be described using probability distributions (based on observations in Supplementary Material 2). The uncertainties of shoreline change projections compare well with those of idealized energetic sandy shorelines exposed to sea-level changes close to the global average^55^. Note that the majority of coasts are expected to be affected by sea-level changes close to or slightly higher than the global average^56^. However, in addition to the uncertainties presented in Fig. 4, there are additional unknowns due to a lack of confidence in the probability distributions themselves^57–59^. These unknowns are called residual uncertainties hereafter, following the terminology of Robinson *et al*.^60^. They are listed in Table 1.

**Table 1:** Residual uncertainties of probabilistic shoreline change projections.

Source of uncertainty	Uncertainties quantification in this study	Residual uncertainties (not quantified in this study)
Future sea-level rise	Probabilistic regional sea-level rise projections^32^	Possibility of rapid melting of ice-sheets^61,65^
Vertical ground motions	Geodetic uncertainties at the permanent GNSS at Cap Ferret^48^	Representativeness of the GNSS records (linear extrapolations in time and space)
Shoreline change variability and trends	Seasonal, interannual and multi-decadal (~50 years) shoreline evolutions	Structure of equation (1); Possible alteration of natural variability modes and or self-organized patterns^89^
Uncertainties of the shoreface and upper shoreface slopes	Observed variability of shoreface and upper shoreface slopes	The actual variability may be larger and the slopes may exceed their current variability in the future
Shoreline evolution modeling framework	Structural uncertainties (2 different modeling approaches)	Limited confidence in both modeling frameworks
Uncertainties of the PCR model	Statistical uncertainties, reflecting the variability of the response of the PCR model to virtual time series of events (Supplementary Material 1 Section 2; Supplementary Fig. 5).	Structural uncertainties: choices made in the different modules of the PCR model, especially the dune erosion module.
Uncertainties of the Bruun rule	Observed variability of shoreface slopes	Limitations of the Bruun rule^43,90^

Sea-level rise projections presented here implicitly assign a very low probability to Antarctic marine ice-cliff instabilities^61^, although the probability that such instabilities occur during the 21^st^ century remains unquantified so far^62^. Sea-level projections considering marine ice-cliff instabilities are significantly higher than those used in this study, as their median attains 1.8 m as early as 2100^62,63^. Our results reflect the fact that according to the sea-level projections of Kopp *et al*.^32^ and those provided by the IPCC^2^, future sea-level rise remains a slow process characterized by a large inertia and involving multi-centennial timescales^64^. Replacing the current reference projections^2,32^ by those derived from DeConto and Pollard^61^ would change coastal impact assessments^65^, so that this residual source of uncertainty would deserve specific attention from an adaptation perspective. Furthermore, the selection of a particular climate change scenario has little impact to future shoreline positions within the probabilistic framework presented in this study (brown area in Fig. 4). This reflects the fact that sea level projections used here^32^ are not differentiated enough according to climate change scenarios to create large differences in shoreline change projections given the uncertainties of other processes (Fig. 1). In these projections, the dynamic contribution of ice in Antarctica is largely independent from greenhouse gas emissions due to the lack of understanding of processes taking place today^66^. However, sea level rise will continue for millennia even if we keep climate warming below the target of the Paris agreement, and the rates of sea level changes over the next centuries will strongly depend on current mitigation policies^64,67^.

Vertical land motions are subject to large residual uncertainties as well: indeed, the analysis of eleven years of continuously recorded GNSS data at Cap Ferret suggests that site #2 is affected by a subsidence of −1.2 ± 0.6 mm/yr^48^, which is qualitatively in agreement with the independent estimate from supplementary levelling data (Source: Aquitaine Coastal Observatory). However, it is unsure that the pointwise information of the permanent GNSS station located at Cap Ferret is representative of the nearby area.

The terms in equation (1) contain some uncertainty as they are estimated based on limited observations of shorelines, and foreshore/upper foreshore slopes (Table 1 and Supplementary Material 1). However, the structure of equation (1) itself can be discussed because it assumes that only sea-level rise will modify the sediment budgets, that the system will remain mostly unaffected by human interventions, and that potential changes of other factors such as waves remain within the error bars of $Tx$ and $Lvar$. As noted in the introduction, an alternative would be to aggregate the contribution of each process known to cause net multi-decadal shoreline changes. These would include the effects of persistent longshore sediment transport gradients, decreased inner shelf sediment supply to the shoreface, sediment losses to the submarine canyon south of Aquitaine and where the longshore drift terminates, and aeolian transport (the impact of reduced sediments from rivers is believed to be negligible in the 4 sites investigated here). In Aquitaine, as in many coastal sites worldwide, these processes remain poorly quantified, suggesting that the actual uncertainties associated with $Tx$ might be larger than those presented in Supplementary Material 3. However, it should be noted that the retrospective simulations of past shoreline positions in the 19^th^ century (in gray), which are known from historical maps, are very sensitive to errors in $Tx$. If we had used erroneous multi-decadal trends, this would results in past shoreline positions hardly reconcilable with this independent piece of evidence. This makes us confident that the multi-decadal trends used in this work are close to the ground truth. Furthermore, current modes of shoreline change variability could be altered by climate change^68,69^. However, climate change models do not indicate that a modification of storm patterns (intensity, trajectories) should be expected in the Bay of Biscay, so that the impacts on wave regimes and surges are expected to remain small compared to those of sea-level rise. Hence, we believe that using equation (1) with the values of $Tx$ and $Lvar$ presented in this study is the best possible approach given the present knowledge on the study site. Rare events such as the unusual 2013/2014 sequence of winter storms suggest that more research is needed in this area^70,71^.

Coastal impact model uncertainties are quantified here using the differences between two well-established models. However, there is no guarantee that this metrics is appropriate to appraise the real variability of possible impacts of sea-level rise on sandy shorelines. Among the two models, the PCR model results, owing the inherent probabilistic approach adopted in the model, incorporate the statistical uncertainty reflecting the variability of profile response to virtual time series of storm events. However, structural uncertainties, due to choices made during the implementation of the PCR model itself, are not quantified. These include uncertainties due to choices made while generating virtual time series of events (Supplementary material 4, section 3), while computing total water level estimates at the coast^72^, and when computing dune recovery and erosion^73^. Based on our knowledge in Aquitaine and on our experience in developing and applying the PCR model elsewhere in the world, we feel that the dune erosion module is potentially the largest source of structural uncertainties^45^ of the PCR model outcome in our particular application. In particular, the dune erosion module^73^ assumes that the dune toe evolves parallel to the slope of the upper shoreface, which remains within the same range over time.

In this article, we concentrated the analysis on quantifiable uncertainties (using the term introduced by Walker *et al*.^74^) without attempting to quantify residual uncertainties. However, we not only treated parametric uncertainties (i.e. uncertainties related to the choice of the values in the model parameters), but also model uncertainties, i.e. structural uncertainties (uncertainties related to the selection in the most appropriate model structure given our problem). The latter uncertainty source is, to our best knowledge, rarely addressed in the literature and falls usually under the category of residual uncertainties.

## Conclusion

Despite limitations, our results show that coastal managers in charge of identifying and implementing appropriate adaptation decisions on sandy beaches need to consider uncertainties of sea-level rise together with those of coastal impact models. In fact, the structure of the two coastal impact models available today implies that slopes used to quantify the impacts of sea level rise may vary from the upper foreshore slopes (as in the surrogate PCR model) to the entire foreshore slopes, i.e., measured over a transect starting at the “depth of closure” and ending at the dune toe. This means that for most of the beaches worldwide, structural uncertainties due to the selection of the coastal impact model can be expected to have large impacts in shoreline change projections.

This statement is especially valid at multi-decadal to centennial timescales, which are the most relevant for land-use planning. Hence, our results call for more research in the area of coastal impact models in order to support coastal adaptation. Over the past decades, sea-level rise has been accelerating^4,5^, and a further acceleration is expected to take place without mitigation of climate change. At the same time, coastal evolution modeling is progressing toward appropriate complexity approaches assimilating coastal observations^20,75^. We speculate that the impacts of sea-level rise on sandy shorelines should be increasingly observable in the coming decades, so that global, long term, repeated, accurate and precise observations of shoreline positions will be especially relevant to support the development of more trustworthy coastal impact models.

## Methods

### Sea-level reconstructions and projections

Sea-level projections are those of Kopp *et al*.^32^ (Fig. 1). These projections provide the probability of future sea-level rise at La Rochelle. By analyzing tide gauge records, Kopp *et al*.^32^ estimate a subsidence at La Rochelle to be −0.55 +/− 0.52 mm/yr. However, this subsidence is within the error bars. Consequently, the null hypothesis of a stable location cannot be ruled out at the 95% confidence level. Furthermore, the permanent GNSS station co-located with the La Rochelle tide gauge indicates that the tide gauge is stable too^48^. Hence, we removed the subsidence estimation in the projections of Kopp *et al*.^32^. We assume that the probabilistic sea-level rise projections at La Rochelle are applicable in Aquitaine once corrected from the local vertical ground motions.

To reconstruct past sea levels in the Bay of Biscay, we averaged yearly records of 15 stations available in the Permanent Service for Mean Sea Level (PSMSL) corrected for local vertical land motions. We rejected 5 additional stations because they displayed anomalous trends (Pointe Saint Gildas, Le Verdon, Pasajes, Santander 2 and Gijon 2). For each station, the local rates of sea-level rise and their uncertainties were computed using a forward-backward Kalman filter^76,77^ as in Rohmer and Le Cozannet^78^. Local vertical land motions were obtained either using permanent GNSS stations from the SONEL database^48^, or, in the absence of GNSS station, using global isostatic adjustment models^79^ available at tide gauge in Jevrejeva *et al*.^80^. Where no GNSS information was available, where the GNSS station was located too far from the tide gauge, or where the GNSS records displayed large step discontinuities or a clear non-linear behavior, we assigned a Gaussian uncertainty of 2 mm/yr (1-sigma) to the vertical land motion value, as obtained from the histogram frequency distribution of all trends in the SONEL database corrected from the global isostatic adjustment. Note that larger subsidence or uplift values would be detectable in the tide gauge sea-level time series. Finally, we computed past mean sea levels and their uncertainties using a weighted least square regression. The method used here assumes that vertical land motions are linear over the timescales considered, and that all stations measure the same signal of mean sea level in the Bay of Biscay.

We assume that vertical land motions at the site #2 are those measured by the GNSS station of the Cap-Ferret. In other sites, as no permanent GNSS with sufficiently long records are available, we modelled the uncertainties due to vertical ground motions through a centered normal distribution with a standard deviation of 2 mm/yr, as obtained from the histogram analysis of all trends computed from the coastal permanent GNSS stations in the SONEL database^33^.

### Coastal impacts models

The Bruun rule quantifies the shoreline retreat in response to sea-level rise as follows^34^:

2 $$\Delta {S}_{slr}=\frac{{\rm{\Delta }}\xi }{\tan (\alpha )}$$

where ${\rm{\Delta }}\xi$ is the cumulated rise of sea level and $\tan (\alpha )$ is the foreshore slope from the depth of closure to the top of the upper shoreface, usually close to 1%^81^.

We implement a variant of the PCR model adapted to the Aquitaine coast and present the results for site #2. Following Ranasinghe *et al*.^35^, we use the Larson *et al*.^73^ formula to compute sediment losses during storms. Furthermore, we parametrize the dune recovery rate in the PCR model using observations in order to minimize this source of uncertainty^35–37^: we estimate gains at the dune toe under calm weather conditions at 25 ± 15 m^3^/m/year, based on an analysis of 9 dune profile campaigns of the Aquitaine Coastal Observatory from 2007 to 2015. Finally, we incorporate tides as suggested in Larson *et al*.^73^. The model inputs are virtual time series of events (surge level, significant wave height, peak period and peak direction, tidal levels, event duration, spacing between events) capturing the statistical dependence between variables, their seasonality and event grouping, which are generated using waves and surges hindcasts^68,82^ (see Supplementary Material 1, section 2).

### Global sensitivity analysis

The Variance-Based Sensitivity Analysis (VBSA) quantifies the contribution of input variables and parameters to the variance of the outcomes of a model^51^ (see Chu-Agor *et al*.^83^; Wong and Keller^84^, Le Cozannet *et al*.^55,85^ for applications of global sensitivity analysis in the area of coastal impacts of sea-level rise). Let us define *f* as the model computing future shoreline change positions. Considering the *n*-dimensional vector ***X*** as a random vector of independent random variables *X*_*i*_ (i = 1,2, …, *n*) (Table 1), then the output *Y* = *f*(***X***) is also a random variable (as a function of a random vector). VBSA determines the part of the total unconditional variance Var(*Y*) of the output *Y* resulting from each input random variable *X*_*i*_. The partial and total variances of *Y* are assessed based on the functional analysis of variance decomposition of *f* ^86^, into summands of increasing dimension (provided that *f* can be integrated). Each of these terms can be evaluated through multidimensional integrals, which can be approximated through Monte-Carlo-based algorithms.

On this basis, the Sobol’ indices (ranging between null and unity) can be defined as:

3 $${S}_{i}=\frac{Var[E(Y|{X}_{i})]}{Var({Y}_{j})}$$

The first-order indices *S*_i_ are referred to as “the main effects of *X*_*i*_” and can be interpreted as the expected proportion of the total variance of the output *Y* (*i.e*. representing the uncertainty in *Y*) that would be removed if we were able to learn the true value of *X*_i_. This index provides a measure of importance (i.e. a sensitivity measure) useful when ranking, in terms of importance, the different input parameters. As a general principle, we attempted to reduce the subjectivity in the representation of our knowledge by relying on the maximum entropy principle for probability law selection^46^. We use the sequential algorithm of Saltelli *et al*.^87^, using the R implementation of the Jansen formula^87,88^, and a total of 200,000 model evaluations per time step, which allows to reduce the errors of the first-order indices below 1%.

### Surrogate of the PCR model

Under stable sea levels, the PCR models behaves as follows: depending on the frequency and intensity of storms and on the initial conditions, the shoreline, identified as the dune toe, moves around an equilibrium shoreline position, toward which the PCR model converges after a transitional phase of 20 to 30 years. As sea level rises, high water levels during storms become more frequent and induce a retreat of the dune toe parallel to the slopes of the upper shoreface^73^, so that sediment blown to the dune by aeolian processes cannot compensate losses and a new equilibrium position is found for the dune toe^35^. The Figure provided in Supplementary Material 5 displays this equilibrium response as a function of sea-level change rates. It shows that its value is close to the amount of sea-level rise divided by the slope of the upper shoreface (See section 4 in Supplementary Material 1). The same equilibrium response is obtained for sea-level scenarios following step functions or parabolic curves, as in the sea-level projections used here^32^. This response, which is here illustrated in the case of site #2, can be explained by the basic principles of the dune erosion model, which assumes that the dune toe evolves parallel to the upper shoreface^73^, while the slope of the upper shoreface itself remains within the same range over time (see Supplementary Material 2). While other dune evolution models may deliver different responses, the more complex model SBEACH has provided similar responses so far^35^.

## Supplementary information

Supplementary information

## Data Availability

Results and codes will be provided in an open archive.
