# Author Correction: Quantifying uncertainties of sandy shoreline change projections as sea level rises

**DOI:** 10.1038/s41598-019-41667-3

**Published:** 2019-04-11

**Authors:** Gonéri Le Cozannet, Thomas Bulteau, Bruno Castelle, Roshanka Ranasinghe, Guy Wöppelmann, Jeremy Rohmer, Nicolas Bernon, Déborah Idier, Jessie Louisor, David Salas-y-Mélia

**Affiliations:** 1 BRGM, 3, av. Claude Guillemin, BP 36009, 45060 Orleans Cedex 2, France; 2 BRGM, French Geological Survey, Pessac, France; 3 CNRS/Univ. Bordeaux, Pessac, France; 4 IHE Delft/University of Twente/Deltares, Delft, The Netherlands; 5 LIENSs, CNRS - Université de La Rochelle, La Rochelle, France; 6 CNRM, Université de Toulouse, Météo-France, CNRS, Toulouse, France

Correction to: *Scientific Reports* 10.1038/s41598-018-37017-4, published online 10 January 2019

This Article contains an error in Figure 4, where the key was omitted. The correct Figure 4 appears below as Fig. 1.

**Figure 1:**
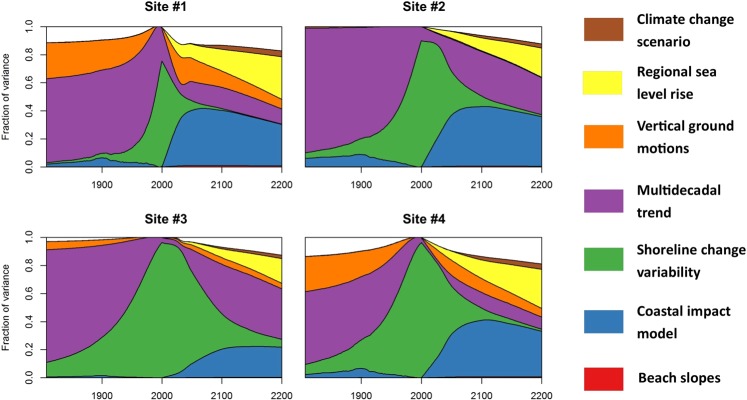
Variance-based global sensitivity analysis of the shoreline change model response as a function of time, for the four selected sites in Aquitaine (see Supplementary Material 1). For each date considered, the curves indicate the fraction of the variance of shoreline change projections that could be removed if input parameters were known (see main text). The effect of interactions between parameters is indicated as well. White areas indicate interactions between parameters, corresponding to shoreline positions, which can be only reached if at least two uncertain parameters deviate from their mean (see Methods). The graph reads as follows: for site #1, by 2200, uncertainties in regional sea-level rise projections (yellow) account for approximately 30% of the variance of shoreline change projections.

