# Supplementary material for: Quantifying uncertainties of sandy shoreline change projections as sea level rises
Source: Sci Rep. 2019 Jan 10;9:42. doi: 10.1038/s41598-018-37017-4 (PMC6328552; doi:10.1038/s41598-018-37017-4)
Supplement: Supplementary file 1 — Supplementary information [file 41598_2018_37017_MOESM1_ESM.docx]

**Quantifying uncertainties of sandy shoreline change projections
as sea level rises**

***Supplementary Material***

Gonéri Le Cozannet^1,*^, Thomas Bulteau^2^, Bruno Castelle^3^, Roshanka Ranasinghe^4^, Guy Wöppelmann^5^, Jeremy Rohmer^1^, Nicolas Bernon^2^, Déborah Idier^1^, Jessie Louisor^1^, David Salas-y-Mélia^6^

1. BRGM, 3, av. Claude Guillemin, BP 36009, 45060 Orleans Cedex 2, France, [g.lecozannet@brgm.fr](mailto:g.lecozannet@brgm.fr), +33 2 38 64 36 14 (Corresponding author)

2. BRGM, French Geological Survey, Pessac, France

3. CNRS/Univ. Bordeaux, Pessac, France

4. IHE Delft/University of Twente/Deltares, The Netherlands

5. LIENSs, CNRS - Université de La Rochelle, France

6 CNRM, Université de Toulouse, Météo-France, CNRS, Toulouse, France

* Contact information:

Dr. Gonéri Le Cozannet

3, avenue Claude Guillemin

45060, Orléans

France

[g.lecozannet@brgm.fr](mailto:g.lecozannet@brgm.fr)

**Supplementary Material 1 – supplementary text**

1. **Coastal settings**

The approach is applied to the open ocean beaches of the French Aquitaine coast (Bay of Biscay). This sandy coast is 230 km long, sparsely urbanized, and constituted by high-energy meso-macrotidal open beaches which are backed by high (generally 15 to 20 m) and wide (generally larger than 100 m) coastal dunes. The surface geology presents no constraints (e.g., non erodible rocks) preventing the shorelines from retreating to the extents simulated by the models. Topographic transects of the beach-dune systems are annually monitored with cm-level precision surveys using differential kinematic Global Positioning System (GPS) since 2008 over more than 50 transects^I^, providing useful data to analyze inter-annual variations of the beach-dune system (dune nourishment by aeolian processes, slopes of the upper shoreface). The DGPS profiles are acquired in spring each year, which may lead to an underestimation of the current variability of upper shorefaces slopes, as they can be larger or smaller in winter compared to spring/summer. The shoreface slopes used in the Bruun rule are based on an analysis of the beach topography (to discern the dune toe) and of the bathymetry (to determine the depth of closure). Estimates of the foreshore and upper foreshore slopes are sensitive to the boundaries chosen to demarcate these zones, with larger uncertainties for upper foreshore slopes. These uncertainties are however smaller than those due to the selection of a particular coastal impact model. Finally, an analysis of past shoreline positions based on remote sensing images allows to analyze shoreline variability and trends at different timescales^47^ (Supplementary Material 4).

Panels B and C in Supplementary Material 4 show that the estimation of multi-decadal trends and variability of shoreline positions are mostly insensitive to the removal of the least reliable shoreline position (1950), suggesting that the evaluation of shoreline change variability and trends are robust against outliers and missing shoreline data. Hence, based on the observations presented in supplementary Material 2, a probability distribution can be proposed to represent the uncertainties of the different processes contributing to shoreline changes included in equation (1) for four contrasted sites (Supplementary Material 2, 3 and 4). We compute $Tx$ and $Lvar$ and their uncertainties by means of a Monte Carlo procedure implementing equation (1), where $Tx$ is estimated by linear regression of the observed shoreline positions and subtracting the effects of sea level rise, while $Lvar$ is estimated based on the residuals.

1. **Method used to generate virtual time series of events**

The PCR model requires simulating virtual time series of the following statistically dependent variables: significant wave height *Hs*, peak period *Tp*, peak direction *θp*, event duration D*,* surge *η*, tidal level *A*, spacing between events *G*. We used wave data from BoBWA_10kH database^70^ (http://bobwa.brgm.fr). The extracted point is located (44.65°N ; -1.45°E) at 51 m depth and the covered period is 1958-2002 at hourly time step. Surges and tidal signal come from a reanalysis covering the period 1979-2009 at 10 min time step^84^. The full temporal simulation proceeds in seven steps:

1. Select independent events from original time series of waves and surge. Following the approach of Gouldby et al. (2014)^II^ we introduced a notional flooding level based on wave setup calculated with the formula of Stockdon et al. (2006)^III^ and surges:

$$FL=setup+ \eta$$

A flooding level time series of 23.66 years is thus created. Independent events are then selected using a Peak-Over-Threshold (POT) approach and appropriate independence temporal criteria (minimum of 1 day between the end of an event and the beginning of the next one, or, if it is not verified, a minimum of 2 days between two consecutive FL peaks). We end up with 473 independent combinations of (*Hs, Tp, θp, D, η*).
2. Fit extreme value distributions (Generalized Pareto Distributions) to wave height *Hs*, surge *η* and storm duration *D* (marginal distributions).
3. Fit the semi-parametric conditional extreme model of Heffernan and Tawn (2004)^IV^ to model dependencies between *Hs*, *D* and *η*.
4. Fit a non-homogenous Poisson distribution to the spacing between storms *G* taking into account event grouping as in Callaghan et al. (2008)^V^ and Luceño et al. (2006)^VI^.
5. Generate times series of combinations of (*Hs, D, η, G*) representing 100 years by means of a Monte-Carlo procedure.
6. Fit the peak wave period and peak direction conditional distributions (conditioned to *Hs*) and complete the synthetic dataset with simulations of *Tp* and *θp*. The same models as in Gouldby et al. (2014)^II^ are used for *Tp* and *θp*.
7. Finally, simulate the tidal level *A* for each synthetic event. To do so, a Saros period (18.6 years) of the tidal signal is duplicated multiple times so that the total length covers the simulated period (100 years). Then, we select the tidal peak closest to each simulated event.

Steps 5 to 7 are repeated multiple times to be able to characterize the statistical uncertainty due to random Monte Carlo simulations. Structural uncertainties are due to choices made during the design of the procedure generating virtual time series of events. This includes choices made when implementing the POT approach, when choosing a model to adjust marginal distributions, when choosing a dependence model between variables, when choosing appropriate thresholds to adjust this dependence model, etc. In each step, we relied on statistical and visual tests and diagnostic tools so that the subjective choice of thresholds selection converges as much as possible to an objective one. For the remaining uncertainties linked to the particular choice of GPD (for marginal distributions) and the H&T model (for dependency), we relied on previously published extensive research on this topic, which makes us comfortable with the use of such models.

1. **Surrogate of the PCR model**

Under stable sea levels, the PCR models behaves as follows: depending on the frequency and intensity of storms and on the initial conditions, the shoreline, identified as the dune toe, moves around an equilibrium shoreline position, toward which the PCR model converges after a transitional phase of 20 to 30 years. As sea level rises, high water levels during storms become more frequent and induce a retreat of the dune toe parallel to the slopes of the upper shoreface^75^, so that sediment blown to the dune by aeolian processes cannot compensate losses and a new equilibrium position is found for the dune toe^35^.

The gray areas in the Figure provided in Supplementary Material 5 show the variability of the PCR model response due to its response to 50 time series of virtual events (see Methods). Dark gray indicates likely shoreline change rates, whose probability is larger than 66% according to the simulations (consistently with the IPCC terminology on uncertainties). Light gray areas provide information regarding higher quantiles, displaying the possible values of very likely and virtually certain shoreline change rates according to the PCR simulations. The standard deviation of the shoreline positions obtained with the PCR model is less than 9 m given a fixed sea level rise step, which is smaller or of the same order than the observed shoreline change variability (8 to 20 m according to the panel B in the Supplementary Material 4). This shoreline change variability is included in equation (1) through the term $Lvar$.

Supplementary Material 5 shows that in a first approximation, the equilibrium response of the PCR model can be emulated by equation (2), where the slopes of the upper shoreface (from 4 to 13%, see Supplementary Material 2) are substituted to the Bruun slopes (from 1.2 to 1.5% in Aquitaine). This is in agreement with previous (unpublished) analyses, undertaken within the context of the PCR model applications reported by previous studies^36,37,38^, in relation to the dependency between final PCR model outputs (i.e. exceedance probability curve of coastline recession) on statistical choices such as the marginal distribution model, joint probability distributions and the various thresholds used therein has proved to be small. Finally, using equation (1) with the Bruun rule or the PCR surrogate model has a negligible computation time, so that it becomes possible to perform a propagation of probabilistic uncertainties and a global sensitivity analysis by means of a quasi-Monte-Carlo procedure.

1. **Additional references of the supplementary material:**

Bulteau T., *et al.* Évaluation de l’impact des tempêtes de l’hiver 2013- 2014 sur la morphologie de la Côte Aquitaine. Rapport final. BRGM/RP-63797-FR, 68 p., 138 fig., 8 tab., 2 ann. <http://www.brgm.fr/sites/default/brgm/projets/oca/RP-63797-FR.pdf> (2014).

Gouldby, B., Méndez, F. J., Guanche, Y., Rueda, A., & Mínguez, R. A methodology for deriving extreme nearshore sea conditions for structural design and flood risk analysis, *Coast. Eng.*, **88**, 15–26 (2014).

Stockdon, H. F., Holman, R. A., Howd, P. A., & Sallenger Jr, A. H. Empirical parameterization of setup, swash, and runup. *Coastal engineering*, *53*(7), 573-588 (2006).

Heffernan, J. E., & Tawn, J. A. A conditional approach for multivariate extreme values. *Journal of the Royal Statistical Society*, **66**, 3, 497-546 (2004).

Callaghan D., Nielsen P., Short A., & Ranasinghe R. Statistical simulation of wave climate and extreme beach erosion*. Coast Eng* **55**(5):375–390 (2008).

Luceño, A., Menéndez, M., Méndez, F. The effect of temporal dependence on the estimation of the frequency of extreme ocean climate events. Proceedings of the Royal Society of London, Series A **462** (2070), 1683–1697 (2006).

Bernon N., *et al.* caractérisation de l’aléa recul du trait de côte sur le littoral de la côte aquitaine aux horizons 2025 et 2050. Rapport final. BRGM/RP-66277-FR, 99p., 48 ill., 16 tab., 2 ann. <http://infoterre.brgm.fr/rapports/RP-66277-FR.pdf> (2016).

Idier, D., Castelle, B., Charles, E., & Mallet, C. Longshore sediment flux hindcast: spatio-temporal variability along the SW Atlantic coast of France. *Journal of Coastal Research*, **65**(sp2), 1785-1790 (2013).

**Supplementary Material 2 – supplementary Table**

Observations and models allowing the computation of probabilistic representation of the uncertainties associated with input parameters in equation (1).

| **Variable** | **Distribution** | **Values** | **Source of information** |
| --- | --- | --- | --- |
| Foreshore slope from the dune toe to the depth of closure | Uniform | 1.2% to 1.5% | Topographic and bathymetric data^VII^ |
| Slope of the upper shoreface (Larson et al., 2004) | Uniform | Site #1 (km 55) : 4% to 10%  Site #2 (km 140) : 6% to 12%  Site #3 (km 165) : 6% to 13%  Site #4 (km 197) : 5% to 13% | Yearly beach profile surveys (DGPS) of the Aquitaine Coastal Observatory^I^ |
| Coastal impact model | Discrete uniform | Bruun or emulation of Ranasinghe | Bruun (1962)^34^  Ranasinghe et al. (2012)^35^ |
| Observed variability of shoreline position ($Lr$) at timescales ranging from events to several decades | Gaussian | Site 1 (km 55) : +/-3.2m  Site 2 (km 140) : +/- 9.4m  Site 3 (km 165) : +/-21m  Site 4 (km 197) : +/-8.5m | Based on past observed multi-decadal shoreline change variability^47^ (see Supplementary Material 4). |
| Linear trend of shoreline evolution at longer timescales (i.e., linear trends of $Lr$) | Gaussian | Site 1 : 0.15+/-0.12m/yr  Site 2 : -0.083+/-0.22m/yr  Site 3 : 1.08+/-0.28m/yr  Site 4 : 0.82+/-0.11m/yr | Based on past observed multi-decadal shoreline change variability (Castelle et al., 2018^47^; see Supplementary Material 4). |
| Vertical ground motion | Gaussian | Site 1 : 0+/-2mm/yr  Site 2 : -1,2±0,6 mm/yr (GNSS)  Site 3 : 0+/-2mm/yr  Site 4 : 0+/-2mm/yr | Based on the permanent GNSS located at Cap-Ferret (site #2). For the other sites, no trend can be computed yet (short records), and the uncertainties of vertical ground motion is based on the mean and standard deviation of all permanent GNSS velocities in the SONEL database^48^ after removal of the effects of the global isostatic adjustment using the ICE-5G model^81^ (see Methods). |
| Past regional sea level rise | Gaussian | Sea Figure 1 | Based on a reconstruction of past sea level in the Bay of Biscay (see Methods) |
| Future regional sea level rise | Non parametric distribution | See Figure 1 | Data of La Rochelle^32^, corrected from vertical ground motions measured with a permanent GNSS station (see methods) |
| Climate change scenario | Discrete uniform | RCP 2.6, 4.5 or 8.5 | Kopp et al., 2014^32^; See Figure 1. |

**Supplementary Material 3 – supplementary Figure**


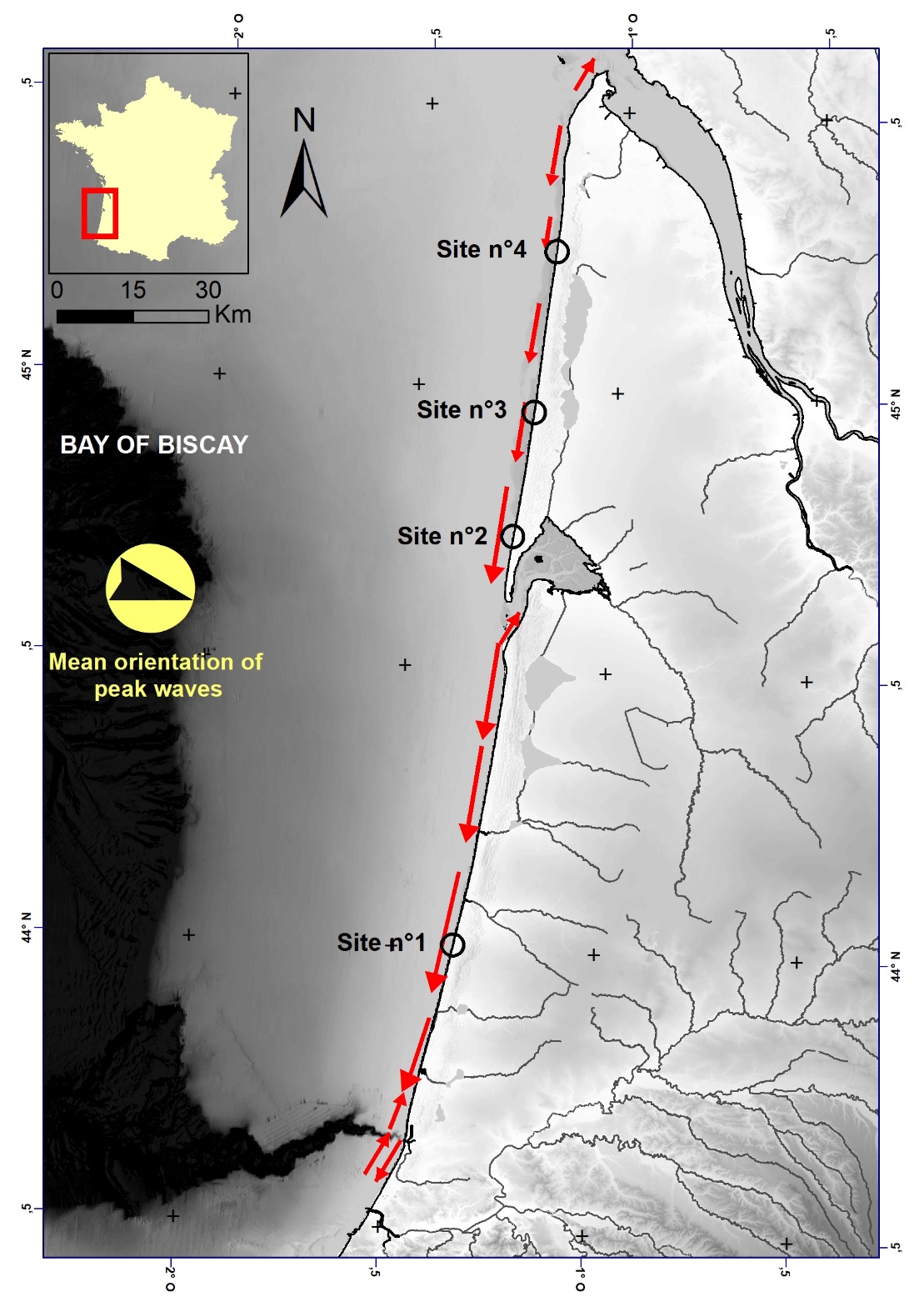


Map showing the location of four selected coastal sites in Aquitaine, the mean orientation of peak waves and the longshore sediment fluxes (Data: Aquitaine Coastal Observatory; Idier et al., 2013^VIII^).

**Supplementary Material 4 – supplementary Figure**


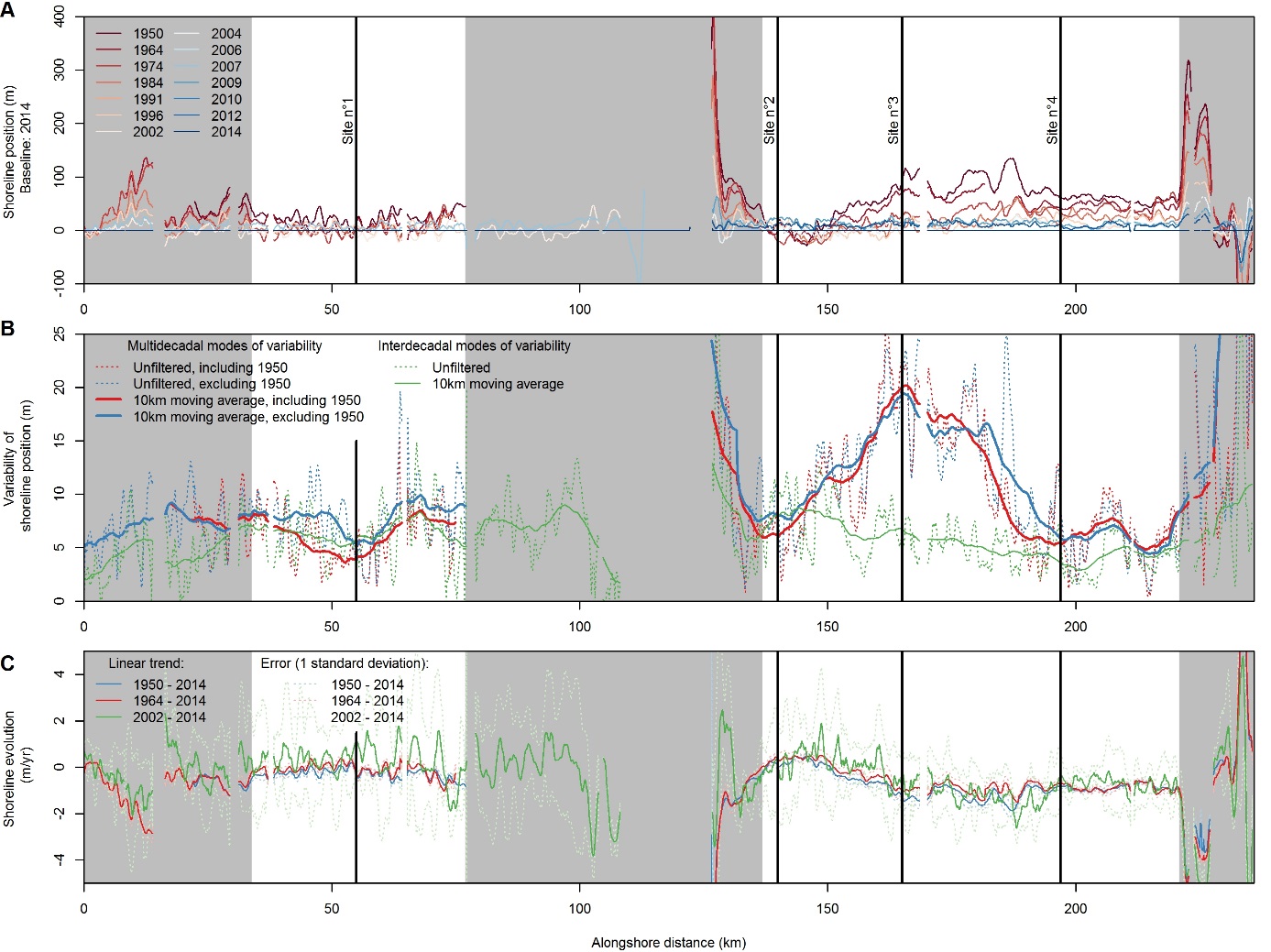


Spatial variability of shoreline position (A), shoreline change variability (B) and trends (C) along the sandy shorelines of Aquitaine. In Panel B, shoreline change variability is estimated as the standard deviation of the de-trended shoreline positions for each transect with at least 3 observations over 3 different decades (blue and red curves) and using all shoreline positions available from 2002 to 2014 (green curves). In areas unaffected by estuarine processes, the average shoreline change variability accounts for ±9.8m around the linear multi-decadal trend if all shorelines (including 1950) are included. The grey areas indicate coastal sites excluded from the analysis, either because they are too close from estuaries or because of the lack of shoreline data (Data: Castelle et al., 2018^47^).

**Supplementary Material 5– supplementary Figure**


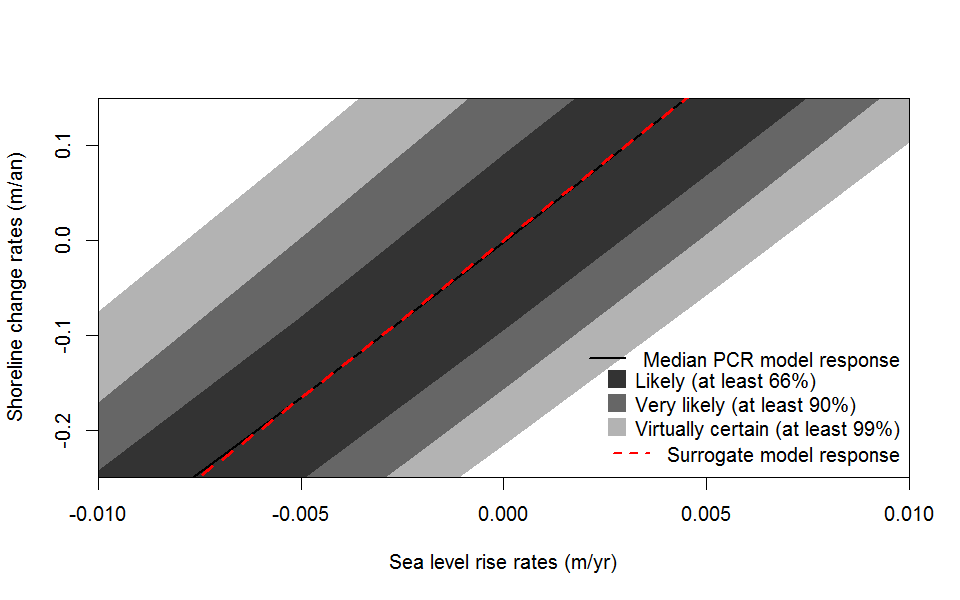


Shoreline change rates and related uncertainties for values of sea level rise ranging from -25mm/yr to +15mm/yr using the PCR model and its surrogate to site #2 (see Methods). Shoreline retreats are computed assuming that the shoreline is at equilibrium at the beginning of the simulations, and that the upper foreshore slope is equal to 3%. The uncertainties of shoreline change rates originate from the use of 50 random time series of 100 years of virtual events as well as from the nourishment of the dune between extreme events (see Supplementary Material 1).

**Supplementary Material 6– supplementary Figure**


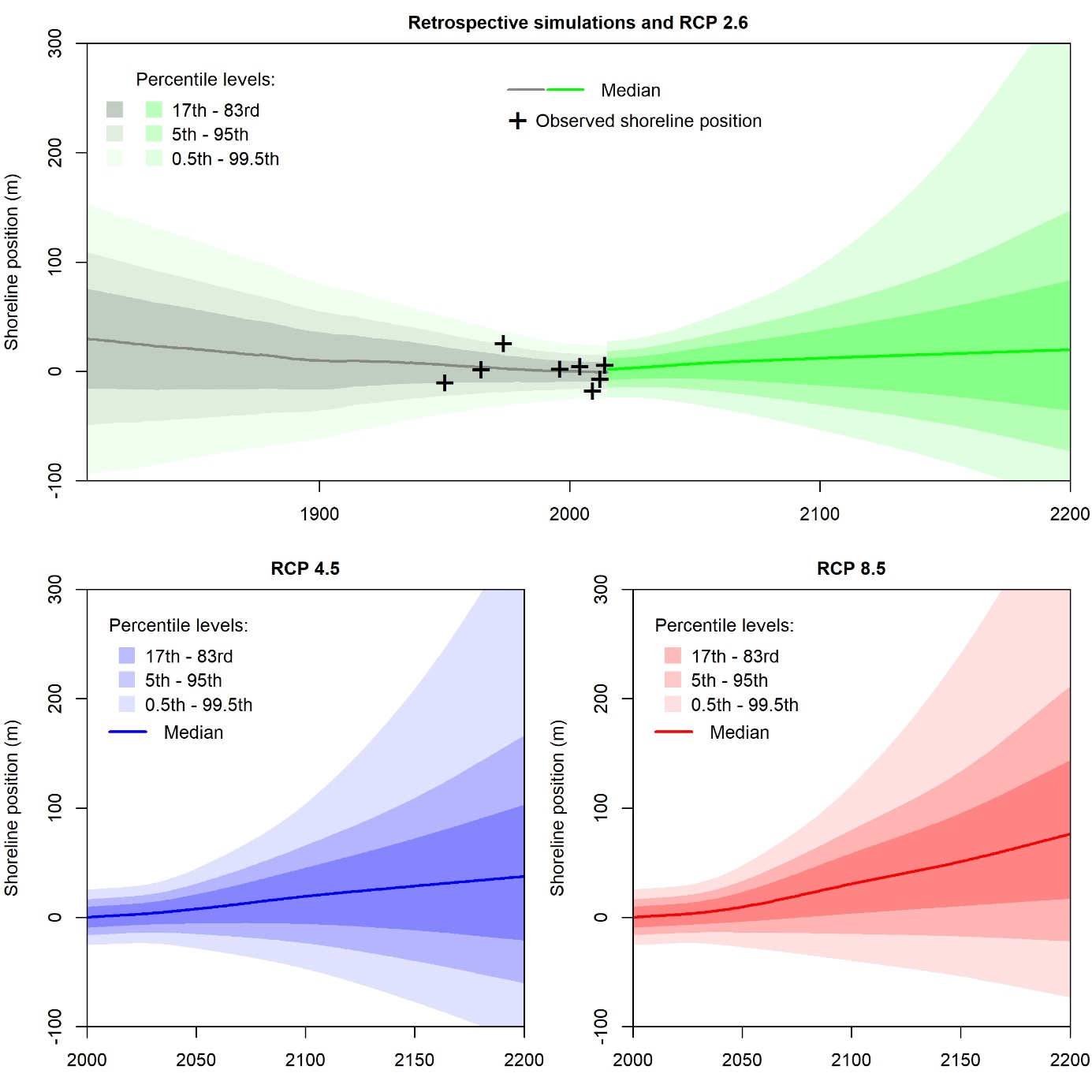


Same as Figure 2, for the site #2.

**Supplementary Material 7– supplementary Figure**


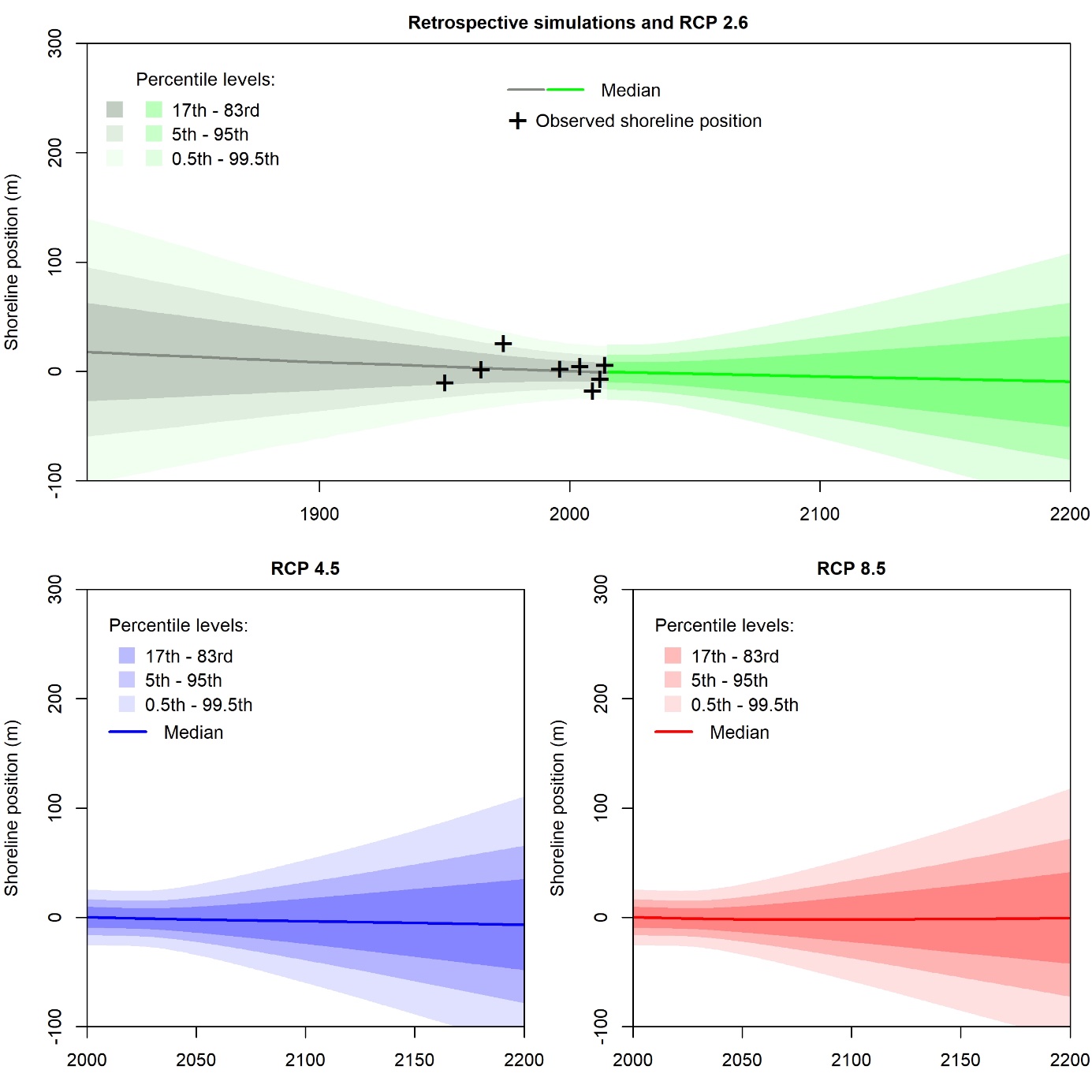


Same as Figure 3, for the site #2

**Supplementary Material 8– supplementary Figure**


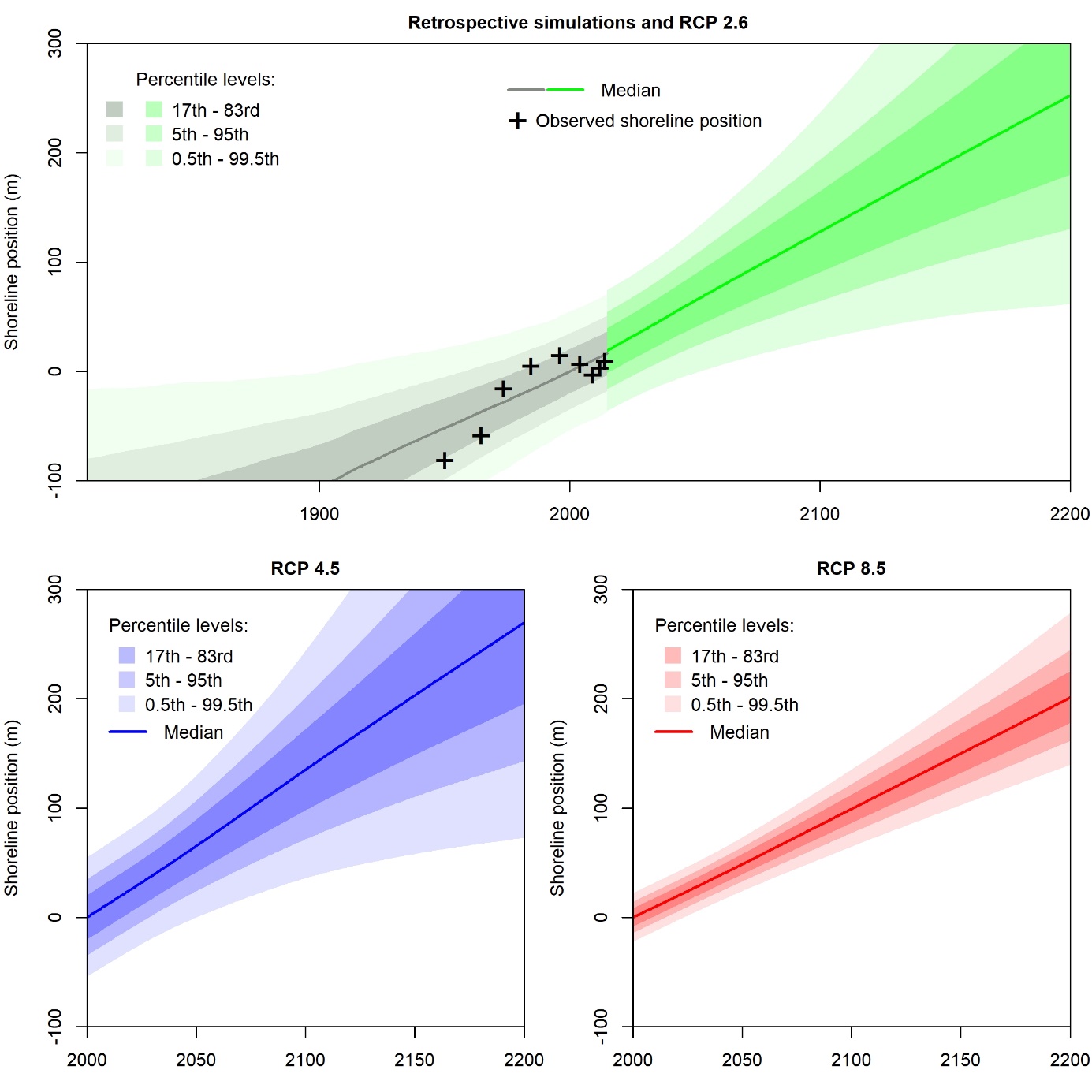


Same as Figure 2, for the site #3.

**Supplementary Material 9– supplementary Figure**


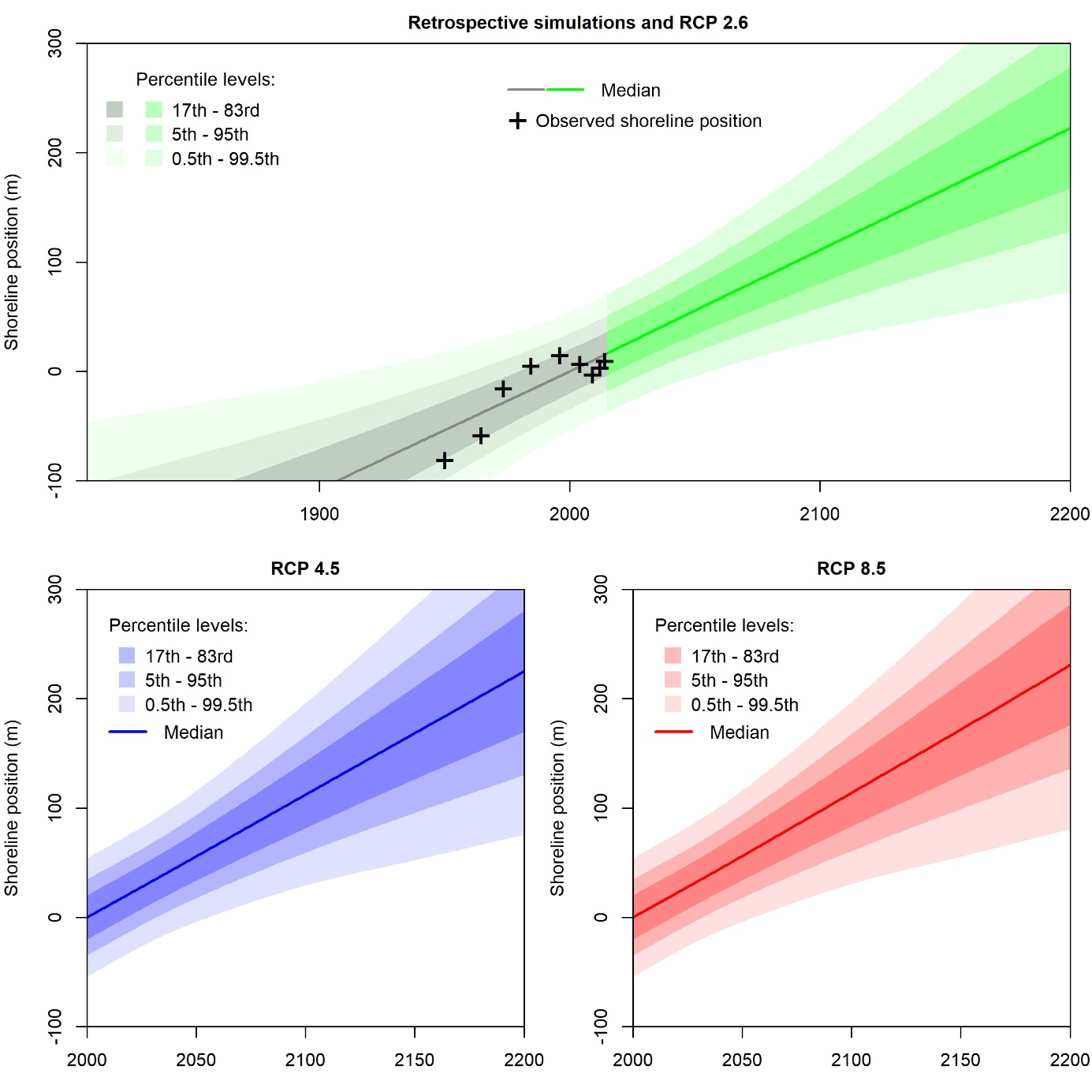


Same as Figure 3, for the site #3.

**Supplementary Material 10– supplementary Figure**


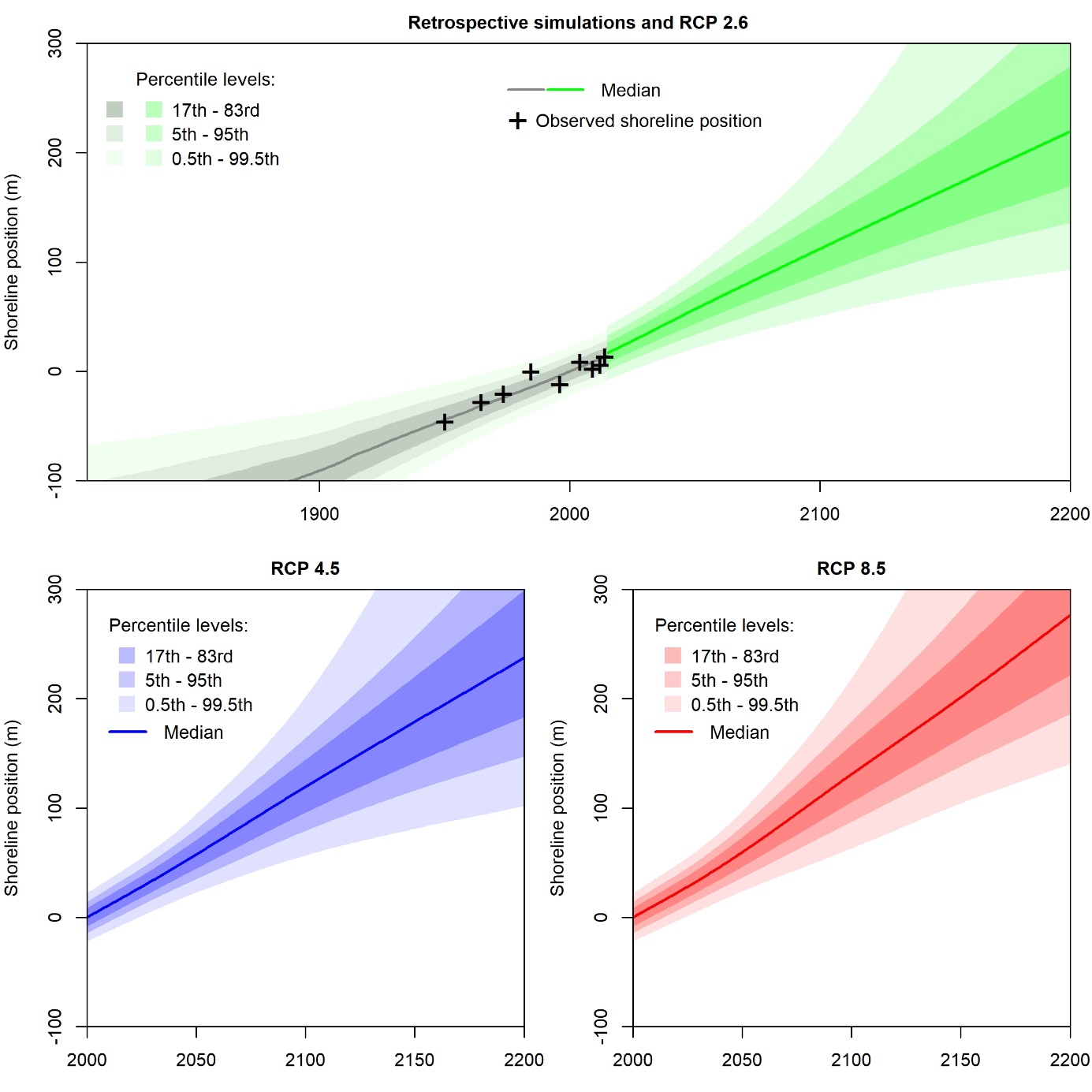


Same as Figure 2, for the site #4.

**Supplementary Material 11– supplementary Figure**


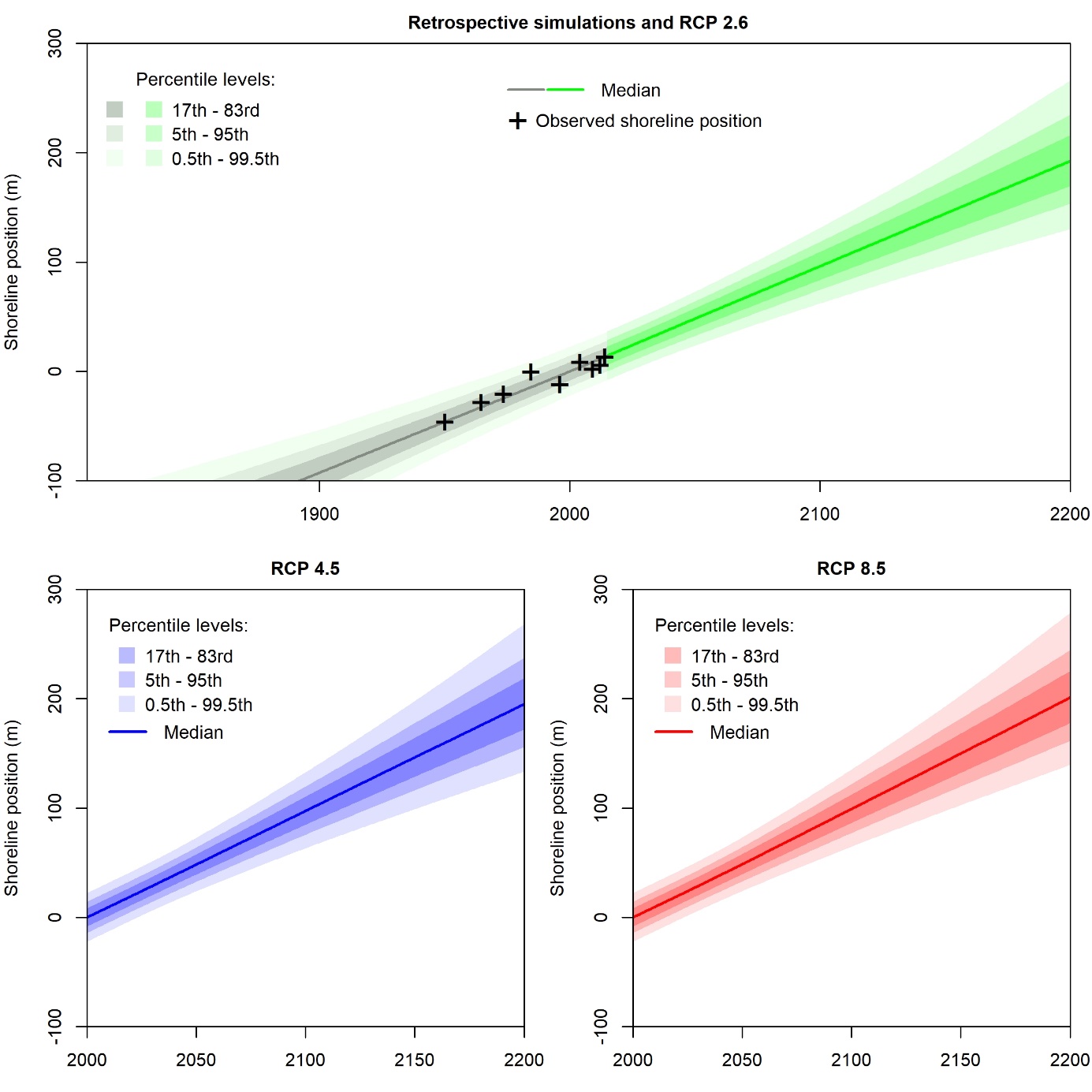


Same as Figure 3, for the site #4.
